# Connecticut

**Published:** 1896-05

**Authors:** 


*Connecticut.* A herd recently tested at Windham showed two-thirds of the number diseased, and the New Haven *News* urges the State to more thorough inspection, and that every citizen demand from his milkman, before accepting the milk, a certificate of inspection, showing the herd free from tuberculosis.

Twenty cows and one bull of the Woodward herd at Thompsonville were recently destroyed on the tuberculin test, and at the autopsies an object-lesson was given to a large number of farmers, cattleowners, and dairymen. Seven animals from three other herds were condemned, destroyed, and, on autopsy, revealed abundant evidence of the disease. In these examinations not a single condemned animal failed to reveal evidence of the disease, though many of them had the appearance of vigor and strong constitutions.

